# Supplementary material for: Efficient Seeding for Error-Prone Sequences with SubseqHash2
Source: bioRxiv. 2024 Jun 3:2024.05.30.596711. Preprint. [Version 1] doi: 10.1101/2024.05.30.596711 (PMC11185578; doi:10.1101/2024.05.30.596711)
Supplement: Supplement 1 [file media-1.pdf]

Supplementary Figures for
“Efficient Seeding for Error-Prone Sequences with SubseqHash2”

Xiang Li[1], Ke Chen[1], and Mingfu Shao[1,2,*]

[1]Department of Computer Science and Engineering, School of Electronic Engineering and Computer Science, The Pennsylvania State University
[2]Huck Institutes of the Life Sciences, The Pennsylvania State University

May 30, 2024

List of Supplementary Figures

1 Probability of hash collision for $n = 20$ and $k = 12, 14$. 2
2 Probability of hash collision for $n = 20$ and $k = 16, 18$. 3
3 Probability of hash collision for $n = 30$ 4
4 The average CPU time in read mapping. 5
5 Precision and sensitivity in read mapping 6
6 True/false coverage in sequence alignment with error rate r= 5%. 7
7 True/false coverage in sequence alignment with error rate $r = 10\%$ 8
8 True/false coverage in sequence alignment with error rate $r = 15\%$, $n = 40$. 9
9 Overlap detection results on *E. coli* and *D. melanogaster* datasets with $n = 40$ 10
10 Overlap detection results on *E. coli* and *D. melanogaster* datasets with $n = 50$ 11
11 Overlap detection results on the *D. melanogaster* dataset with $n = 60$ 12

*Correspondence should be addressed to mxs2589@psu.edu.

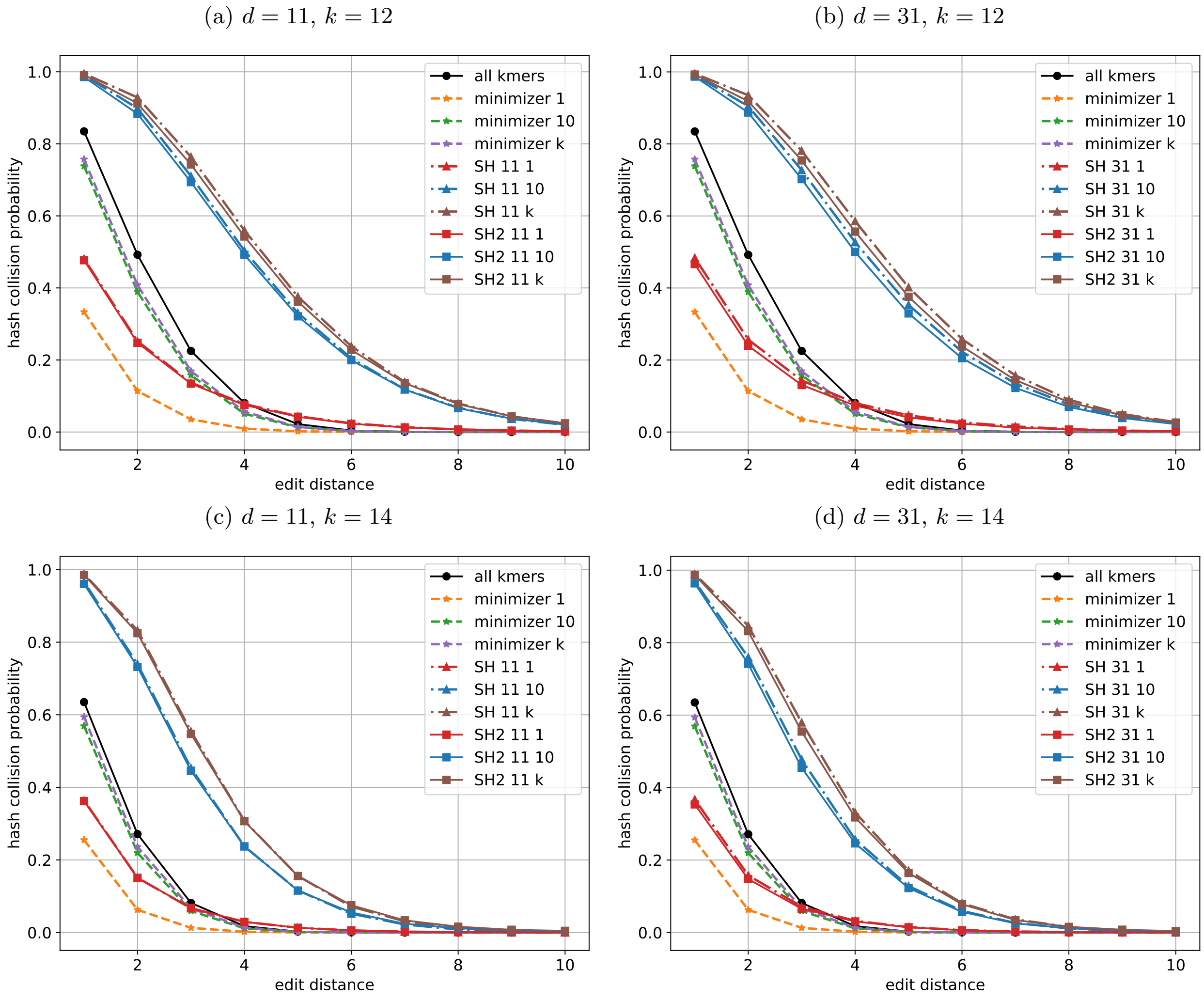

Figure 1: The probability of hash collision, estimated using simulations, for different seeding methods with $n = 20$ and $k = 12$, 14.

(a) $d = 11,\ k = 16$

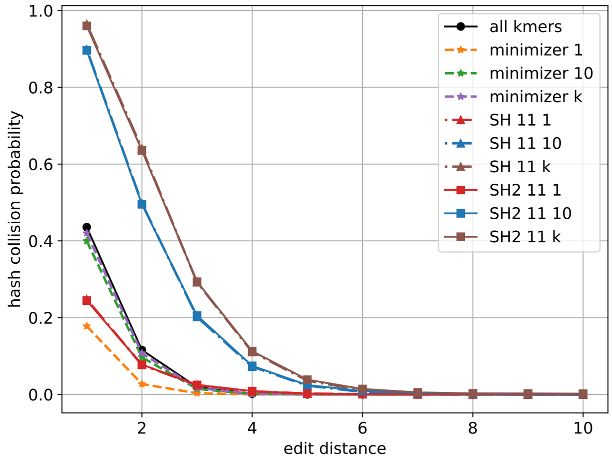

(b) $d = 31,\ k = 16$

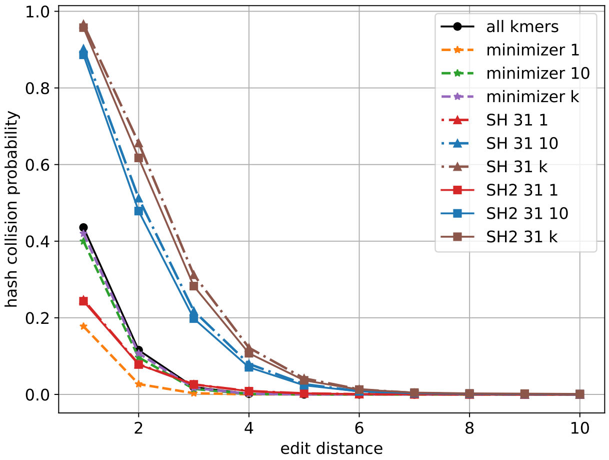

(c) $d = 11,\ k = 18$

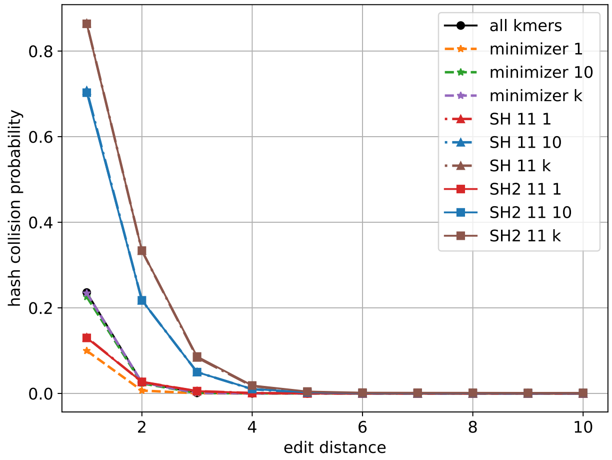

(d) $d = 31,\ k = 18$

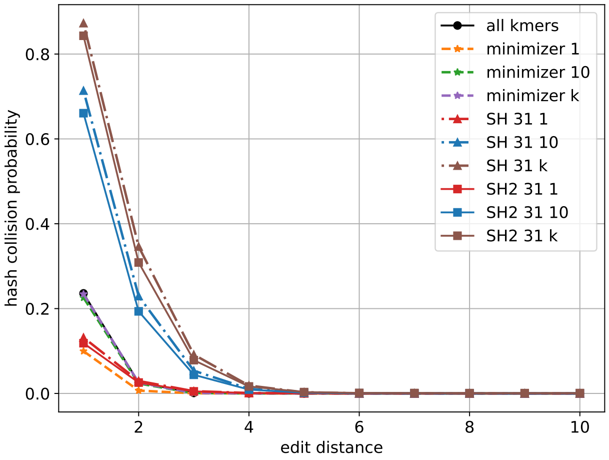

Figure 2: The probability of hash collision, estimated using simulations, for different seeding methods with $n = 20$ and $k = 16, 18$.

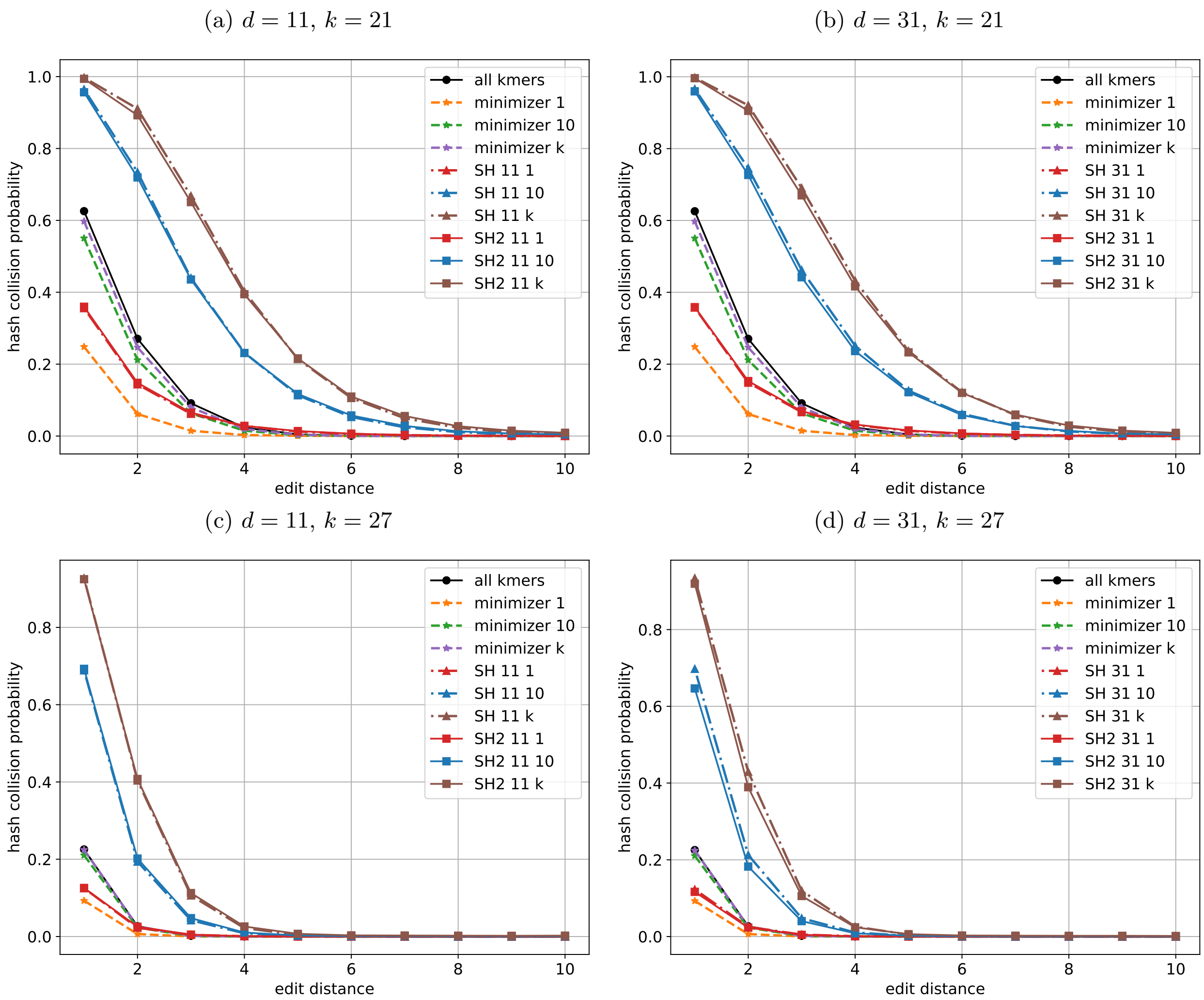

Figure 3: The probability of hash collision, estimated using simulations, for different seeding methods with $n = 30$ and $k = 21, 27$.

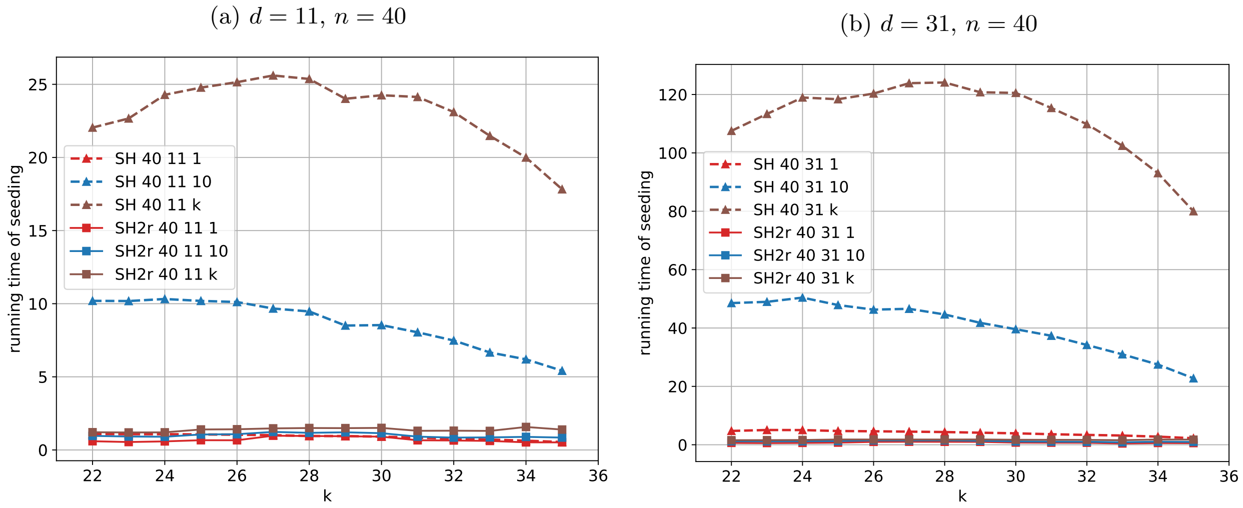

Figure 4: The average CPU time (second) of SubseqHash and SubseqHash2 per read with $n = 40$.

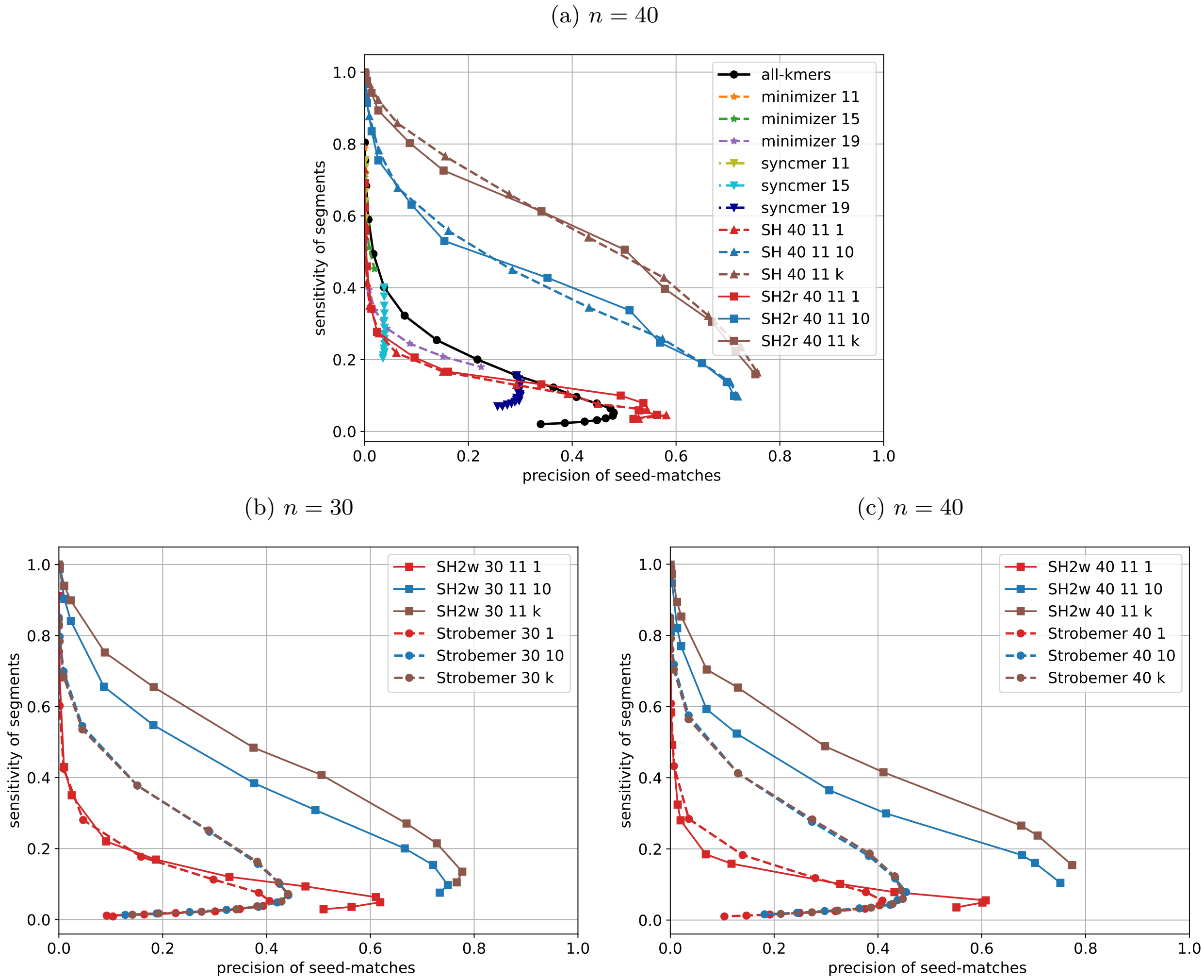

Figure 5: The average precision of seed-matches and sensitivity of segments with different seeding methods with $n = 40$.

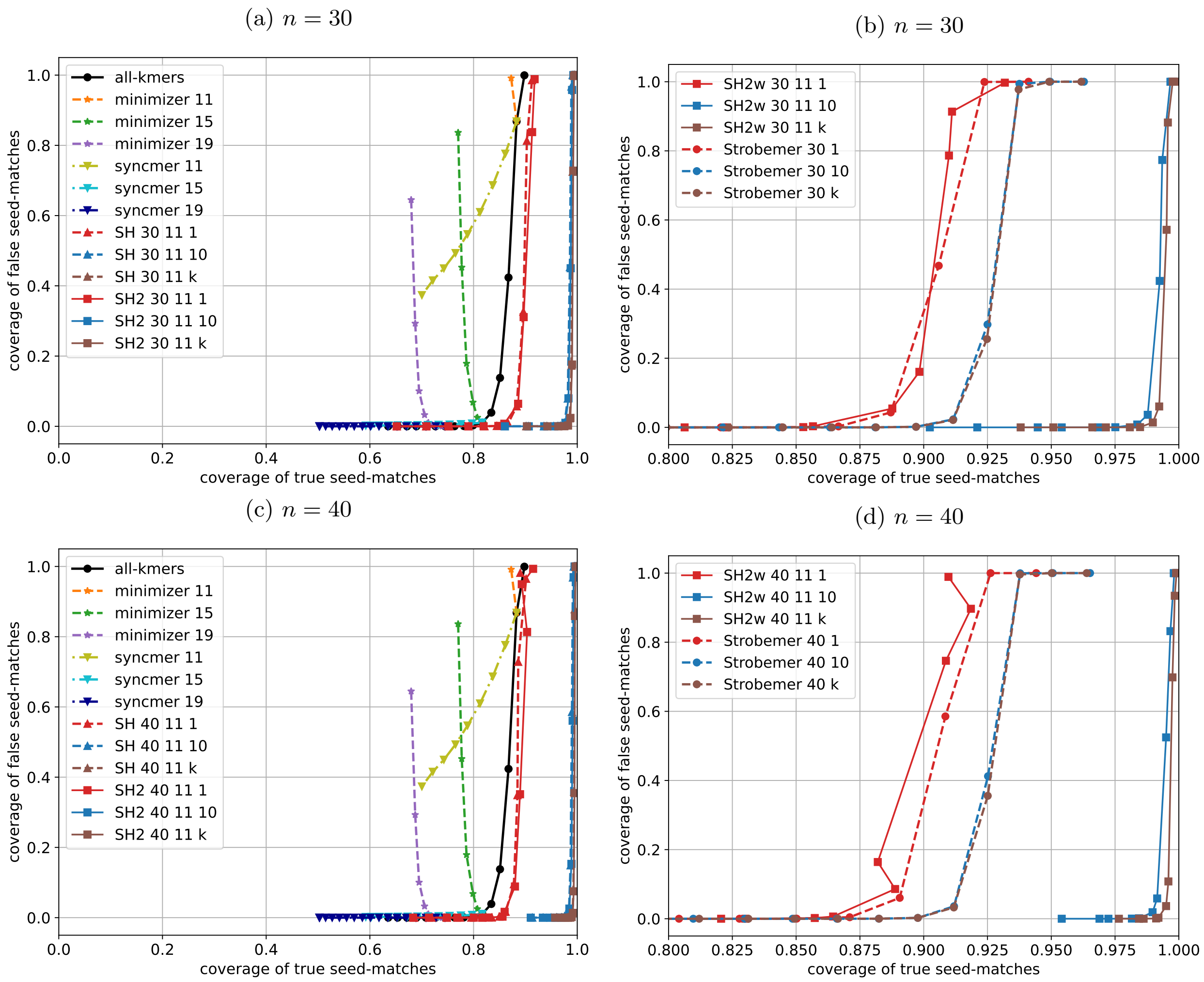

Figure 6: The true and false coverage of different seeding methods in sequence alignment with error rate $r = 5\%$, $n = 30, 40$. Figure (a) and (c) compare SubseqHash2 with all-kmers, minimizer, syncmer, and SubseqHash. Figure (b) and (d) compare SubseqHash2w with Strobemer.

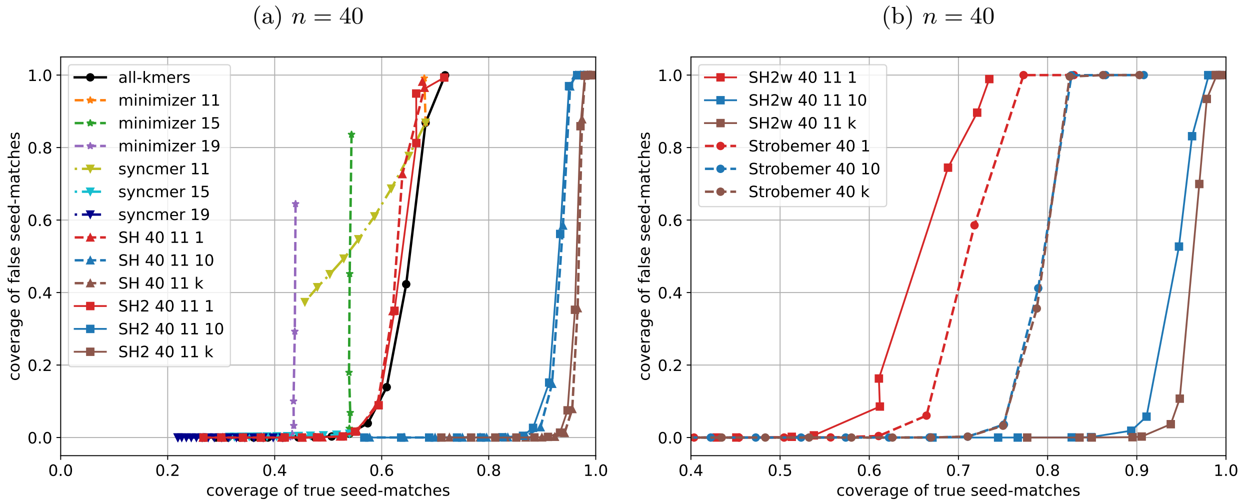

Figure 7: The true and false coverage of different seeding methods in sequence alignment with error rate $r = 10\%$, $n = 40$. Figure (a) compares SubseqHash2 with all-kmers, minimizer, syncmer, and SubseqHash. Figure (b) compares SubseqHash2w with Strobemer.

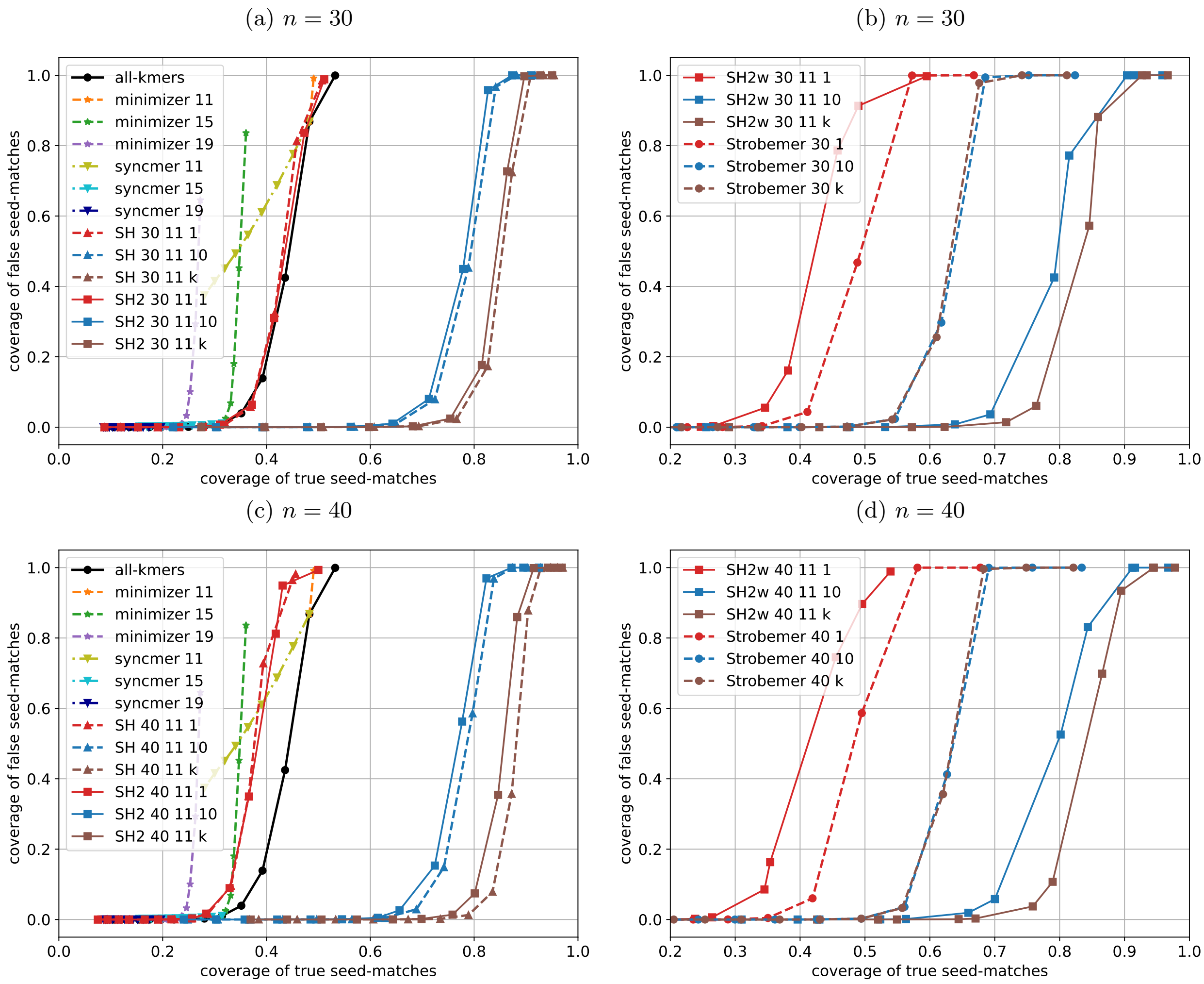

Figure 8: The true and false coverage of different seeding methods in sequence alignment with error rate $r = 15\%$, $n = 30$, 40. Figure (a) and (c) compare SubseqHash2 with all-kmers, minimizer, syncmer, and SubseqHash. Figure (b) and (d) compare SubseqHash2w with Strobemer.

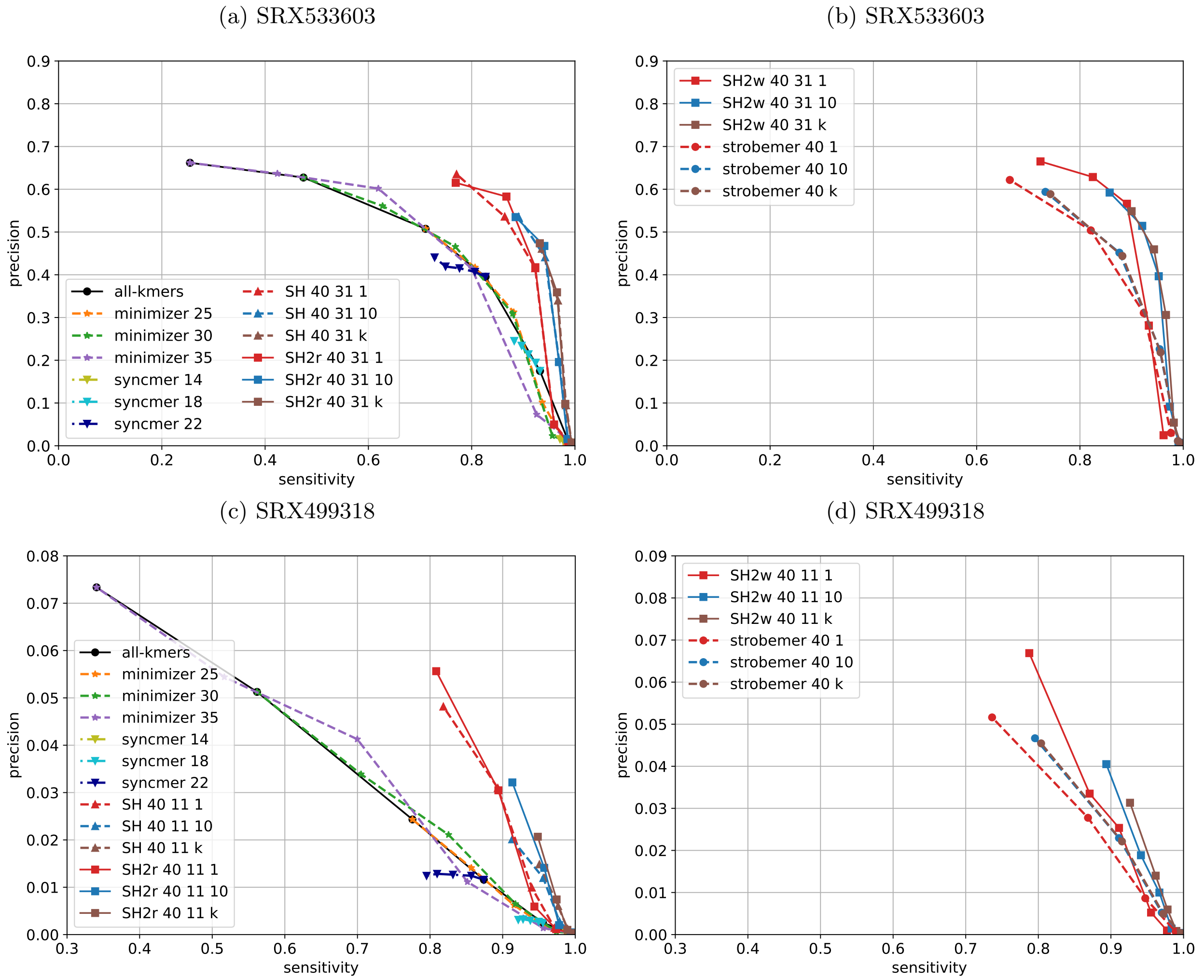

Figure 9: Overlap detection results on 10,000 reads sampled from the the *E. coli* SRX533603 and *D. melanogaster* SRX499318 dataset with $n = 40$. Figure (a) and (c) compare SubseqHash2r with all-kmers, minimizer, syncmer, and SubseqHash. Figure (b) and (d) compare SubseqHash2w with Strobemer.

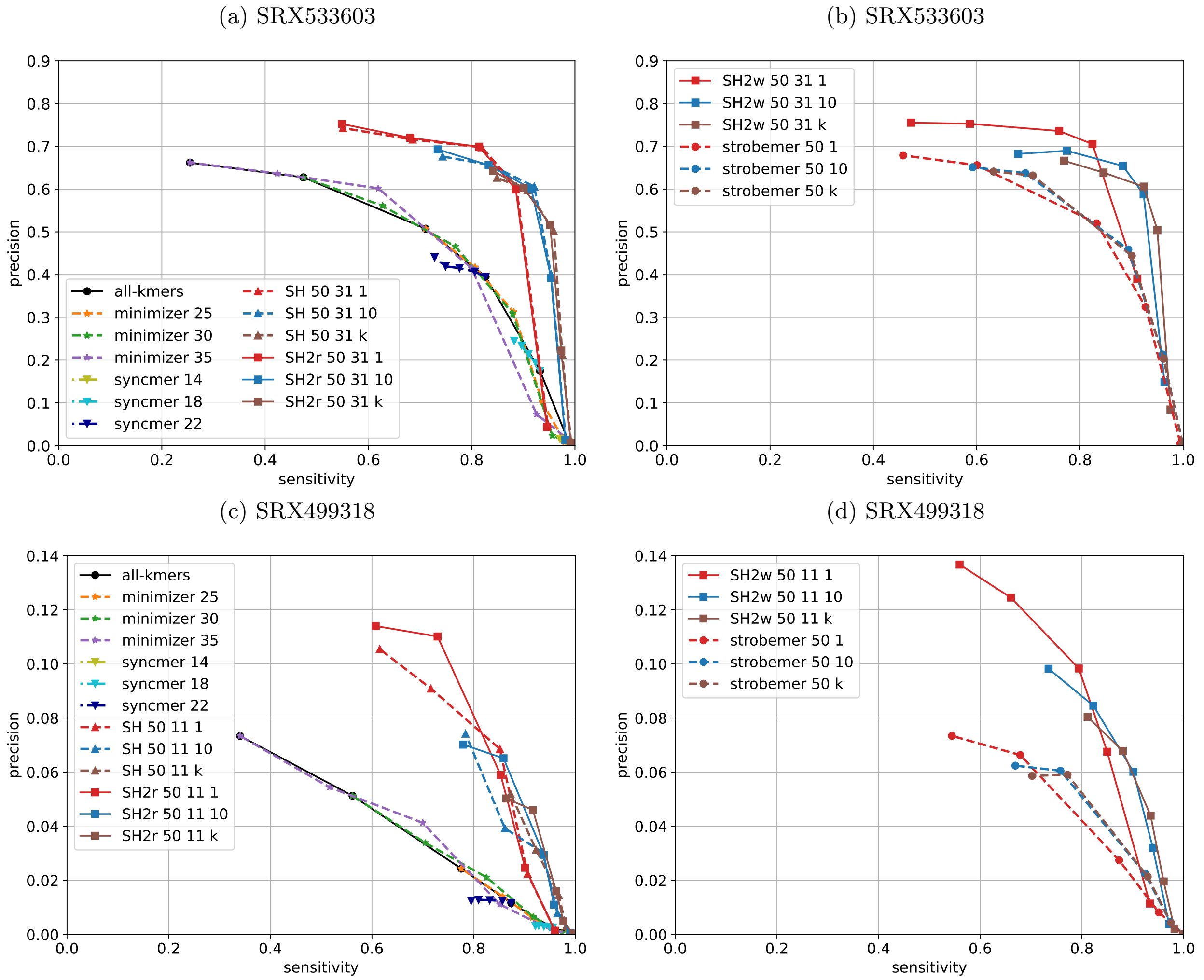

Figure 10: Overlap detection results on 10,000 reads sampled from the the *E. coli* SRX533603 and *D. melanogaster* SRX499318 dataset with $n = 50$. Figure (a) and (c) compare SubseqHash2r with all-kmers, minimizer, syncmer, and SubseqHash. Figure (b) and (d) compare SubseqHash2w with Strobemer.

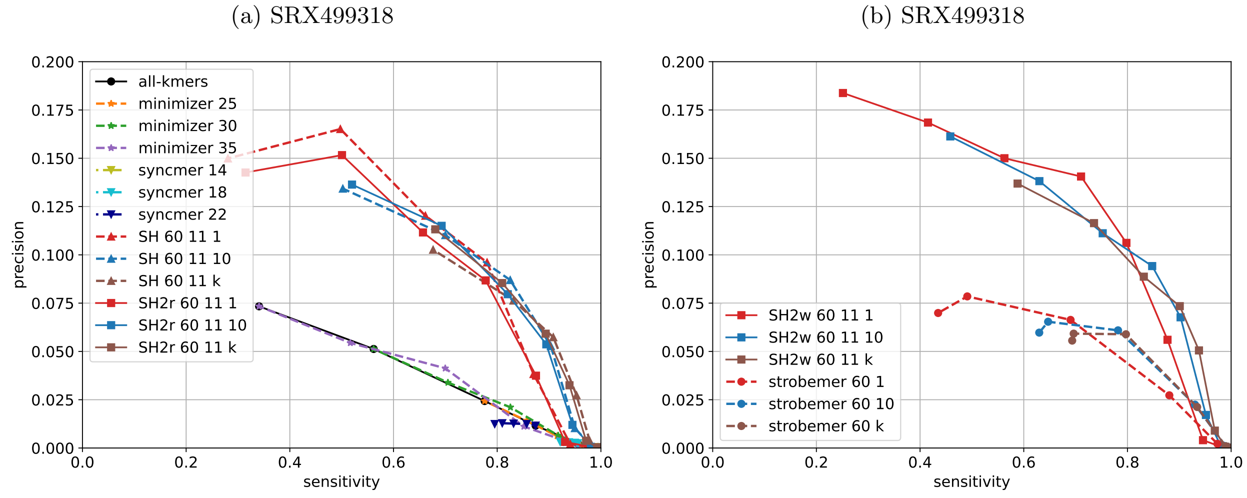

Figure 11: Overlap detection results on 10,000 reads sampled from the the *D. melanogaster* SRX499318 dataset with $n = 60$. Figure (a) compares SubseqHash2r with all-kmers, minimizer, syncmer, and SubseqHash. Figure (b) compares SubseqHash2w with Strobemer.
